# Drusen characteristics of type 2 macular neovascularization in age-related macular degeneration

**DOI:** 10.1186/s12886-020-01651-2

**Published:** 2020-09-25

**Authors:** Daniel Ahmed, Martin Stattin, Anna-Maria Haas, Alexandra Graf, Katharina Krepler, Siamak Ansari-Shahrezaei

**Affiliations:** 1 Karl Landsteiner Institute for Retinal Research and Imaging, Vienna, Austria; 2 Department of Ophthalmology, Rudolf Foundation Hospital, Juchgasse 25, 1030 Vienna, Austria; 3 Center for Medical Statistic, Informatics, and Intelligent Systems, Medical University of Vienna, Spitalgasse 23, 1090 Vienna, Austria; 4 Department of Ophthalmology, Medical University of Graz, Auenbruggerplatz 1, 8036 Graz, Austria

**Keywords:** Age-related macular degeneration, Type 2 macular neovascularization, Drusen, Subretinal drusenoid deposits

## Abstract

**Background:**

Type 2 macular neovascularization (MNV) is supposed to be a rare condition in age-related macular degeneration (AMD). The main purpose of this study was to assess accompanying factors of type 2 MNV in AMD.

**Methods:**

Retrospective data analysis of eyes previously diagnosed with neovascular AMD in a tertiary eye care center (Medical Retina Unit, Rudolf Foundation Hospital, Vienna, Austria) between June 2008 and December 2017. Drusen subtypes, fibrosis, atrophy and subfoveal choroidal thickness (SFCT) of both eyes in patients with type 2 MNV lesions were categorized based on multimodal imaging.

**Results:**

Type 2 MNV was diagnosed in 27 (3.2%) of 835 eyes (749 patients). Drusen characteristics in type 2 MNV were observed as followed: drusen < 63 μm in 2 eyes (7.4%), drusen ≥63 μm in 10 eyes (37%), subretinal drusenoid deposits (SDD) in 8 eyes (29.6%), cuticular drusen in 2 eye (7.4%) and no drusen were evident in 10 eyes (37%). Drusen distribution in 23 fellow eyes was detected as followed: drusen < 63 μm in 2 eyes (8.7%), drusen ≥63 μm in 9 eyes (39.1%), SDD in 5 eyes (21.7%), cuticular drusen in 1 eye (4.3%) and no drusen were evident in 9 eyes (39.1%). Mean SFCT was 140 ± 49 μm in affected eyes and 152 ± 41 μm in the fellow eyes. Patients with drusen or SDD were significantly younger (mean 70.88 ± 6.85, *p* = 0.04) than patients without deposits (mean 77.40 ± 5.74).

**Conclusions:**

Type 2 MNV remains a rare entity in AMD. It was frequently seen in the absence of drusen, a hallmark of AMD. These findings contribute to the heterogeneity of phenotypes related to pure type 2 lesions.

## Background

Early and intermediate age-related macular degeneration (AMD) are defined by the existence of drusen, pigmentary abnormalities or extrafoveal atrophy [1, 2]. Various types of drusen such as soft, hard, cuticular or subretinal drusenoid deposits (SDD) – also referred to as reticular pseudodrusen - confirm with the diagnosis of AMD [3]. Drusen are typically located between the retinal pigment epithelium (RPE) and the Bruch’s membrane, whereas SDD are found in the subretinal space [4–7]. Small drusen (< 63 μm) are considered normal aging changes, while medium (≥63 - < 125 μm) to large drusen (≥125 μm) are associated with an increased risk for the development of late stage disease. Advanced AMD refers to either neovascular AMD (nAMD) or subfoveal geographic atrophy (GA) [1]. Spaide et al. suggested the term macular neovascularization (MNV) more adequate to sum up all neovascular entities in AMD including type 3 [8, 9]. Therefore, the author proposed a new classification system encompassing extracellular deposits as well as the subfoveal choroidal thickness (SFCT) to integrate all aspects of the disease. Type 1 MNV originates from the choroid and remains exclusively underneath the RPE. Polypoidal choroidal vasculopathy has recently been described as aneurysmal neovascularization type 1 and represents a subtype of this entity [10]. A mixed type neovascularization is composed of new choroidal vessels growing in more than one layer. A vascular network only present in the subretinal space is referred to as type 2 MNV. In contrast, type 3 neovascularization - also called retinal angiomatous proliferation (RAP) - reflects a distinct form of nAMD with an intraretinal origin and hence different pathophysiology [11].

While type 1 MNV is the most common subtype in nAMD followed by type 3 and mixed type MNV, the appearance of type 2 MNV is a rare condition [12, 13]. So far, drusen characteristics were mainly investigated in type 1 MNV [1, 2, 14–16]. Type 2 and mixed type MNV with a preponderance towards type 2 – formerly known as classic or predominantly classic choroidal neovascularization - were observed in 11.5–24.6% in the era of FA alone [17–20]. SD-OCT imaging led to a deeper knowledge of the neovascular anatomy as well as the aggregation of extracellular debris [11]. A combination of FA and OCT reduced the percentage of type 2 MNV to 0.8–9% according to the most recent studies with a focus on deposits and choroidal thickness in the latter one [12, 13].

Accompanying factors of type 2 MNV remain still unclear. The main purpose of this study was to gain a better understanding of the characteristics associated with type 2 MNV in AMD based on multimodal imaging.

## Methods

### Patient selection

This retrospective observational case series included treatment- naive patients with nAMD, diagnosed in our tertiary retina center (Medical Retina Unit, Department of Ophthalmology; Rudolf Foundation Hospital Vienna; Karl Landsteiner Institute for Retinal Research and Imaging) between 2008 and December 2017. The study adhered to the tenets of the Declaration of Helsinki. Patients signed an informed consent to participate anonymously in a clinical study. Consenting patients were initially diagnosed and classified into anatomic subtypes of neovascular lesions based on fundus examination by slit-lamp biomicroscopy (Haag- Streit AG, Bern, Switzerland) and multimodal imaging including near-infrared fundus reflectance (IR), blue-peak fundus autofluorescence (BAF) images, spectral-domain (SD) optical coherence tomography (OCT), high resolution fluorescein angiography (FA) and indocyanine green angiography (ICGA). Exclusion criteria were neovascularizations of different entities (p.e. idiopathic, myopic, posttraumatic, uveitic, dystrophic or secondary to pachychoroid diseases) as well as a subfoveal atrophy or a fibrosis at the time of recruitment. The fellow eye was examined for additional information on the drusen distribution and also excluded in case of a neovascularization, subfoveal atrophy or fibrosis. Macular neovascularization secondary to age-related degeneration was concluded if deposits or any other causative origin were absent in both eyes.

### Image interpretation

The data records of patients with type 2 MNV were independently analyzed by two medical retina specialists based on all available images. Type 2 MNV was diagnosed in case of an early leakage and the absence of speckled hyperfluoresence in FA [12]. Multiple SD-OCT scans through the lesion area were conducted to assign the layer according to the subtype classification relative to the RPE. If 95% or more of the lesion was judged by FA and OCT to be type 2 MNV, then it was categorized as type 2 MNV. ICGA differentiated the neovascular lesion as a well-defined network with a circumscribed halo in early phases from other types like polypoidal lesions or hot spots mandatory for type 3 MNV. Moreover, ICGA was helpful to distinguish between neovascularization related to AMD vs. a secondary neovascularization masquerading AMD.

### Characteristics

**Fig. 1:**
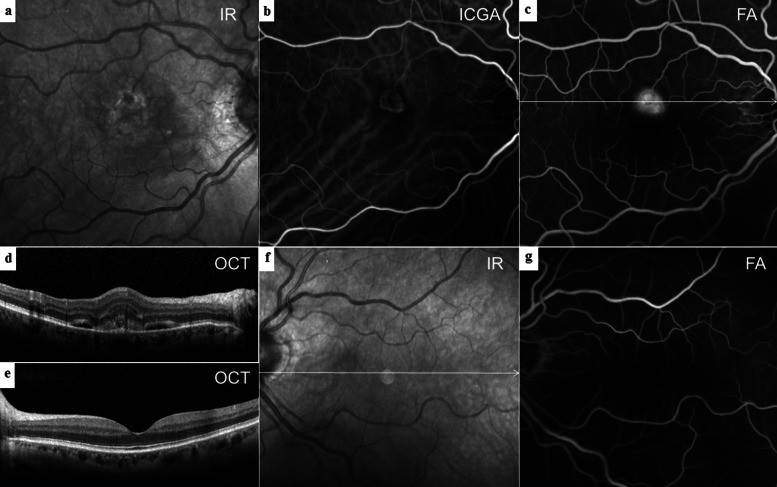
Multimodal imaging of type 2 MNV, Drusen ≥63 μm and SDD. **a** Drusen ≥63 μm (arrowheads) displayed in IR. **b** FA with early MNV leakage and drusen staining (scan line). **c** ICGA revealed a circumscribed MNV besides choroidal vessels under speckled hypocyansecence. **d** SD-OCT B-scan section through the lesion illustrated soft drusen (arrowheads) and a thin SFCT (109 μm). **e** Drusen > 63 (white arrowhead) and SDD (black arrowheads) visualized in the patient’s fellow eye as demonstrated in IR and (**f**) soft drusen staining in late FA

Greyscale variations in IR images on high resolution angiography and OCT scans indicated the existence of soft drusen (Fig. 1a). SDD commonly appeared as hyporeflective dots (Fig. 1e) or ribbons but could also occur as hyperreflective spots surrounding the perifoveal region, termed midperipheral SDD [21].

**Fig. 2:**
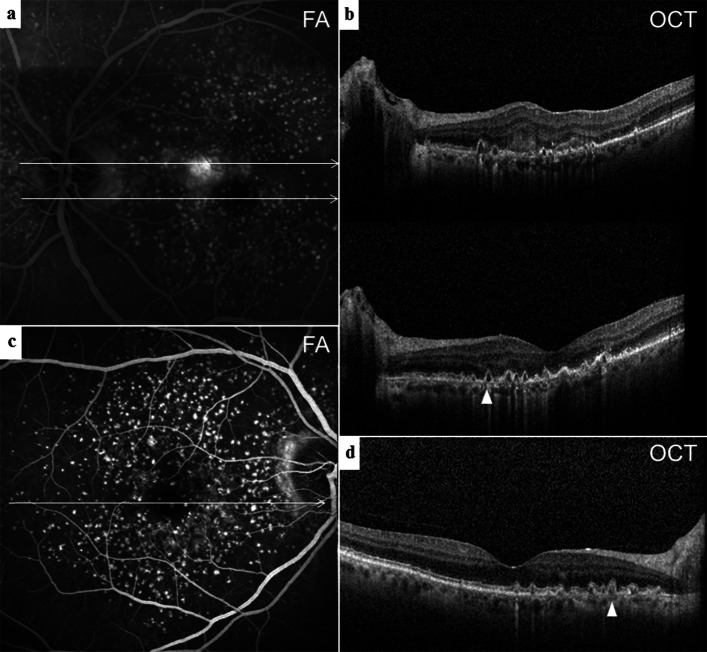
Multimodal imaging of type 2 MNV and cuticular drusen. **a** FA with MNV leakage next to a hyperfluorescent “starry sky” distribution corresponding to the saw tooth configuration (arrowhead) of (**b**) SD-OCT B-scan sections (upper) through the lesion with some hyperreflective material in the subretinal space and (lower) through the foveal umbo. **c** FA demonstrated a similarly clustered posterior pole in the fellow eye with (**d**) multiple elevated pigment epithelium peaks in OCT B-scans typical for cuticular drusen (arrowhead)

In BAF images, soft, hard and cuticular drusen appeared hypoautofluorescent in the center with an annulus of increased fundus autofluorescence [22, 23]. A reticular hypoautofluorescent pattern was typical for SDD [24]. Different staining levels in FA depend on the binding of dye to polar lipids with a higher proportion of fibronectin in contrast to neutral lipids with little adherence [25]. Soft drusen showed mild hyperfluorescence on FA (Fig. 1b, f) but hypofluorescence on ICGA (Fig. 1c), while hard drusen were often hyperfluorescent in both dye applications. SDD were hypofluorescent or not visible on FA and ICGA (Fig. 1b, c, f) [6, 7, 26–28]. The hyperfluorescent “starry sky” appearance on FA was considered as typical for cuticular drusen (Fig. 2a, c) [29]. OCT images were investigated to locate the layer and measure the size of retinal debris. Soft drusen (Fig. 1d), hard drusen or cuticular drusen (Fig. 2b, d) were identified as deposits below or within the RPE, whereas SDD were described as subretinal accumulation of material overlying the RPE zone, forming sharp, broad or rounded elevations [6]. A horizontal diameter of less than 63 μm was defined as threshold to distinguish small from larger drusen. The Spectralis software allowed for a correlation of the topography in SD-OCT scans and 30° en-face images. Drusen phenotypes, SDD, fibrosis or atrophy were identified and listed separately if co-existence occurred. Another senior clinical advisor without affiliation to the study concept was consulted in the case of grading disagreement.

SFCT was measured manually within SD-OCT scans as the greatest vertical distance between the RPE or the Bruch’s membrane and the sclerochoroidal interface.

### Statistics

A univariate logistic regression model was performed for each factor (gender, age, SFCT) potentially influencing the occurrence of drusen or SDD on the affected eye. The same analyses were performed to investigate the fellow eye. All calculations were executed using R, release 3.3.3. Diagrams were developed by Microsoft Excel (Microsoft Corporation, Redmond, WA) and figures composed by Photoshop CC 14.0 (Adobe Systems Incorporated, San Jose, CA).

## Results

Data records of 835 eyes in 749 consecutive patients with nAMD were reviewed for MNV distribution. Type 1 MNV was diagnosed in 658 eyes (78.8%), type 2 MNV in 27 eyes (3.2%), type 3 MNV in 75 eyes (9%), mixed type MNV in 51 eyes (6.1%) and polypoidal MNV in 24 eyes (2.8%).

**Fig. 3:**
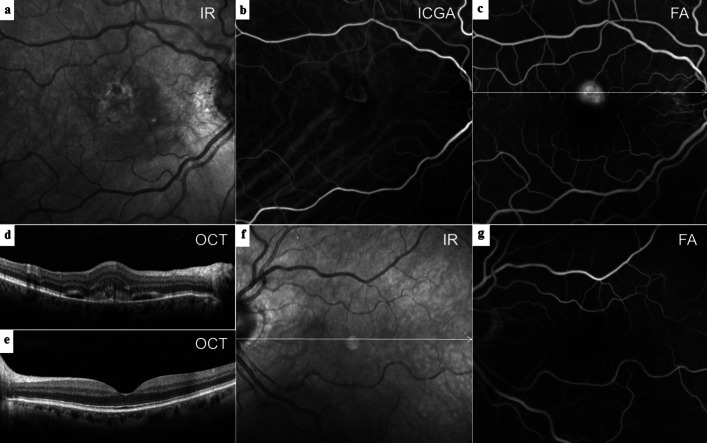
Multimodal imaging of type 2 MNV without deposits. **a** Central hyperreflectivity surrounding a hyporeflective dot induced by the MNV lesion without evidence for drusen or subretinal drusenoid deposits in IR. **b** Early ICGA of the well-defined type 2 MNV. **c** Late FA showing MNV leakage (**d**) 30° SD-OCT B-scan through the lesion with subretinal hyperreflective material next to fluid without outer retina irregularity. **e** No deposits in the fellow eye as demonstrated in a 30° SD-OCT B-scan, (**f**) IR imaging or (**g**) FA

27 eyes of 27 patients with type 2 MNV could be enrolled for further analysis. Type 2 MNV was present in 11 (41%) female and 16 (59%) male patients with a mean age of 73.3 ± 7.1 standard deviation (SD) years (range: 57–86 years). Overall, 17 eyes (63%) compromised by type 2 MNV presented drusen or SDD, leaving 10 eyes (37%) without any extracellular debris (Fig. 3). Drusen or SDD were present in 14 of 23 fellow eyes (60.9%). The distribution of drusen subtypes and their proportions was displayed in Table 1. Patients with drusen or SDD in the neovascular eye were significantly younger (mean 70.88 ± 6.85, *p* = 0.04) than patients without deposits (mean 77.40 ± 5.74). Sex was not significantly associated with the appearance of drusen or SDD. The mean SFCT was 140 ± 49 μm in the neovascular eyes and 152 ± 41 μm in the fellow eyes. It had no significant impact on the occurrence of drusen or SDD. Extrafoveal atrophy was evident in 1 fellow eye (4.3%). Four fellow eyes were excluded either due to neovascularization (1 eye with mixed type MNV) or fibrosis (3 eyes).

**Table 1:** Distribution of characteristics in type 2 macular neovascularization

	Neovascular eyes	Non-neovascular fellow eyes
No drusen	10 (37%)	9 (39.1%)
Drusen < 63 μm	2 (7.4%)	2 (8.7%)
Drusen ≥63 μm	10 (37%)	9 (39.1%)
Cuticular drusen	2 (7.4%)	1 (4.3%)
Subretinal drusenoid deposits (SDD)	8 (29.6%)	5 (21.7%)
Dot SDD	3 (11.1%)	2 (8.7%)
Ribbon SDD	1 (4%)	1 (4.3%)
Dot and ribbon SDD	4 (15%)	2 (8.7%)
Extrafoveal atrophy	0 (0%)	1 (4.3%)
Subfoveal atrophy	0 (0%)	0 (0%)
Fibrosis	0 (0%)	3 (13%)
Total number of eyes	27	23

## Discussion

In this retrospective cohort analysis of 835 eyes complicated by nAMD, only 27 (3.2%) were compromised by type 2 MNV. Drusen or SDD - the hallmark of AMD - were absent in a representative number of 10 (37%) neovascular eyes and 9 (39.1%) fellow eyes. Interestingly, age was the only accompanying factor in a substantial part of patients with pure type 2 lesions. Eyes compromised by this subtype and co-existing deposits were significantly related to younger age in contrast to eyes without additional signs of AMD. It is considered a typical neovascular subtype in entities such as high myopia, inflammatory chorioretinopathies or angioid streaks. Idiopathic choroidal neovascularization was originally described as a focal or type 2 lesion in patients younger than 50 years by Hu et al. in 1995 [30]. These findings suggest a more heterogenic picture of patients compromised by this phenotype of AMD.

Spaide recently proposed a new classification of AMD based on soft drusen, pachydrusen and SDD with predictive capabilities regarding a further development to late stage disease [8]. The appearance of soft drusen might lead to any type of MNV as well as GA. Eyes with SDD - particularly these with dot SDD - could progress to type 3 or type 2 MNV, whereas GA is typically associated with confluent SDD. We were not able to relate SDD subtypes to type 2 MNV. Only one other group focused primarily on clinical characteristics of type 2 lesions in nAMD [13]. 8 of 694 patients expressed this type of neovascularization with 7 of them (88%) possessing SDD and thin choroids. Deposits must have been documented by multimodal imaging before the development of MNV. The authors concluded that the low incidence of type 2 MNV could be attributed to the strict inclusion criteria. Drusen regression with subsequent formation of MNV or GA has already been described [31, 32]. Also, SDD might regress and convert into atrophy over time [33]. We were looking for extracellular material at the onset of neovascular changes and initially excluded eyes with subfoveal atrophy. In concordance to our data, Wilde et al. found no difference in the occurrence of nAMD subtypes and SDD [34]. Another paper published by Marsiglia et al. could not correlate SDD with type 2 MNV significantly 1 year earlier [35]. They investigated the association between MNV subtypes and clinical findings of the non-neovascular fellow eye in patients with unilateral nAMD. SDD and thin SFCT were more frequently seen in fellow eyes of patients with type 3 MNV, whereas type 1 MNV was associated with a decreased odds ratio for SDD and a thin SFCT. We carefully monitored the SFCT of both the affected and the fellow eyes of patients with type 2 MNV and compared eyes with drusen and SDD to eyes without retinal deposits. The term pachydrusen referred to pachychoroid according to Spaide and evolved preferentially into type 1 MNV or polypoidal choroidal vasculopathy [8]. Choroidal thickness measured max. 259 μm in eyes with type 2 MNV, as per definition pachychoroid-associated drusen could not be detected. Moreover, SFCT was not significantly related to extracellular material in the fellow eye. SDD were classified as visible in OCT B-scans but no significant pattern could be assessed in either eye. Drusen or SDD were absent in 9 of 23 fellow eyes (39.1%). Abugreen et al. related neovascular subtypes to AMD severity of the fellow eye and found comparable numbers [36]. No features of AMD were exhibited in 10 of 23 fellow eyes (43.5%) in patients compromised by classic lesions in 2003.

Limitations of this study include the retrospective evaluation of deposits in previously acquired images and their available information. Peripheral SDD or drusen could have been missed as the primary focus of interest was the macular area pictured by Heidelberg’s Spectralis 30° images. Certain questionnaires would be more likely to be answered in a prospective study design. Other MNV subtypes were not analyzed in detail, which impaired the study’s comparative nature. On the other hand, MNV anatomy was examined by multimodal imaging in a large cohort of 835 consecutive patients. Twenty-seven eyes with type 2 MNV in AMD are considered as this study’s strengths. Two thirds of type 2 MNV were found in the presence of soft or hard drusen and SDD. One third of eyes without deposits was frequently related to older age, suggesting a more heterogenic picture of this phenotype.

## Conclusions

The herein presented data contribute to a better understanding of the anatomical features in this rarely found nAMD subtype. AMD is not necessarily confined to the existence of extracellular deposits. Other biological pathways in AMD may lead to the development of a neovascularization limited to the subretinal space.

## Data Availability

The datasets used and/or analyzed during the current study are available from the corresponding author on reasonable request.

## Abbreviations

MNV: Macular neovascularization
AMD: Age-related macular degeneration
SFCT: Subfoveal choroidal thickness
SDD: Subretinal drusenoid deposits
RPE: Retinal pigment epithelium
nAMD: Neovascular AMD
GA: Geographic atrophy
IR: Infrared fundus reflectance
BAF: Blue-peak fundus autofluorescence
SD: Spectral-domain
OCT: Optical coherence tomography;
FA: Fluorescein angiography
ICGA: Indocyanine green angiography
IMNV: Idiopathic macular neovascularization

## References

[1] Age-Related Eye Disease Study Research Group (2001). A randomized, placebo-controlled, clinical trial of high-dose supplementation with vitamins C and E, beta carotene, and zinc for age-related macular degeneration and vision loss: AREDS report no. 8. Arch Ophthalmol.

[2] Ferris FL, Wilkinson CP, Bird A, Chakravarthy U, Chew E, Csaky K (2013). Clinical classification of age-related macular degeneration. Ophthalmology..

[3] Finger RP, Chong E, McGuinness MB, Robman LD, Aung KZ, Giles G (2016). Reticular Pseudodrusen and their association with age-related macular degeneration: the Melbourne collaborative cohort study. Ophthalmology..

[4] Zweifel SA, Spaide RF, Curcio CA, Malek G, Imamura Y (2010). Reticular pseudodrusen are subretinal drusenoid deposits. Ophthalmology.

[5] Zweifel SA, Imamura Y, Spaide TC, Fujiwara T, Spaide RF (2010). Prevalence and significance of subretinal drusenoid deposits (reticular pseudodrusen) in age-related macular degeneration. Ophthalmology..

[6] Spaide RF, Curcio CA (2010). Drusen characterization with multimodal imaging. Retina (Philadelphia, Pa).

[7] Khan KN, Mahroo OA, Khan RS, Mohamed MD, McKibbin M, Bird A (2016). Differentiating drusen: Drusen and drusen-like appearances associated with ageing, age-related macular degeneration, inherited eye disease and other pathological processes. Prog Retin Eye Res.

[8] Spaide RF, IMPROVING THE, AGE-RELATED MACULARDEGENERATIONCONSTRUCT. A new classification system. Retina (Philadelphia, Pa). 2017. 10.1097/IAE.0000000000001732 28557901

[9] Spaide RF, Jaffe GJ, Sarraf D, Freund KB, Sadda SR, Staurenghi G (2020). Consensus nomenclature for reporting Neovascular age-related macular degeneration data: consensus on Neovascular age-related macular degeneration nomenclature study group. Ophthalmology..

[10] Dansingani KK, Gal-Or O, Sadda SR, Yannuzzi LA, Freund KB (2018). Understanding aneurysmal type 1 neovascularization (polypoidal choroidal vasculopathy): a lesson in the taxonomy of “expanded spectra” - a review. Clin Exp Ophthalmol.

[11] Freund KB, Zweifel SA, Engelbert M (2010). Do we need a new classification for choroidal neovascularization in age-related macular degeneration?. Retina (Philadelphia, Pa).

[12] Jung JJ, Chen CY, Mrejen S, Gallego-Pinazo R, Xu L, Marsiglia M (2014). The incidence of neovascular subtypes in newly diagnosed neovascular age-related macular degeneration. Am J Ophthalmol.

[13] Naysan J, Jung JJ, Dansingani KK, Balaratnasingam C, Freund KB (2016). Type 2 (subretinal) neovascularization in age-related macular degeneration associated with pure reticular pseudodrusen phenotype. Retina (Philadelphia, Pa).

[14] Klein R, Klein BEK, Knudtson MD, Meuer SM, Swift M, Gangnon RE (2007). Fifteen-year cumulative incidence of age-related macular degeneration: the beaver dam eye study. Ophthalmology..

[15] Silva R, Cachulo ML, Fonseca P, Bernardes R, Nunes S, Vilhena N (2011). Age-related macular degeneration and risk factors for the development of choroidal neovascularisation in the fellow eye: a 3-year follow-up study. Ophthalmologica..

[16] Abdelfattah NS, Zhang H, Boyer DS, Rosenfeld PJ, Feuer WJ, Gregori G (2016). Drusen volume as a predictor of disease progression in patients with late age-related macular degeneration in the fellow eye. Invest Ophthalmol Vis Sci.

[17] George S, Cooke C, Chakravarthy U (2010). Exudative AMD subtypes and eligibility for treatment with ranibizumab. Eye (Lond).

[18] Cohen SY, Creuzot-Garcher C, Darmon J, Desmettre T, Korobelnik JF, Levrat F (2007). Types of choroidal neovascularisation in newly diagnosed exudative age-related macular degeneration. Br J Ophthalmol.

[19] Olsen TW, Feng X, Kasper TJ, Rath PP, Steuer ER (2004). Fluorescein angiographic lesion type frequency in neovascular age-related macular degeneration. Ophthalmology..

[20] Freund KB, Yannuzzi LA, Sorenson JA (1993). Age-related macular degeneration and choroidal neovascularization. Am J Ophthalmol.

[21] Suzuki M, Sato T, Spaide RF (2014). Pseudodrusen subtypes as delineated by multimodal imaging of the fundus. Am J Ophthalmol.

[22] Balaratnasingam C, Cherepanoff S, Dolz-Marco R, Killingsworth M, Chen FK, Mendis R (2018). Cuticular Drusen: clinical phenotypes and natural history defined using multimodal imaging. Ophthalmology..

[23] Delori FC, Fleckner MR, Goger DG, Weiter JJ, Dorey CK (2000). Autofluorescence distribution associated with drusen in age-related macular degeneration. Invest Ophthalmol Vis Sci.

[24] Smith RT, Chan JK, Busuoic M, Sivagnanavel V, Bird AC, Chong NV (2006). Autofluorescence characteristics of early, atrophic, and high-risk fellow eyes in age-related macular degeneration. Invest Ophthalmol Vis Sci.

[25] Pauleikhoff D, Zuels S, Sheraidah GS, Marshall J, Wessing A, Bird AC (1992). Correlation between biochemical composition and fluorescein binding of deposits in Bruch’s membrane. Ophthalmology..

[26] Arnold JJ, Quaranta M, Soubrane G, Sarks SH, Coscas G (1997). Indocyanine green angiography of drusen. Am J Ophthalmol.

[27] Prenner JL, Rosenblatt BJ, Tolentino MJ, Ying G-S, Javornik NB, Maguire MG (2003). Risk factors for choroidal neovascularization and vision loss in the fellow eye study of CNVPT. Retina (Philadelphia, Pa).

[28] Mimoun G, Soubrane G, Coscas G (1990). Macular drusen. J Fr Ophtalmol.

[29] Gass JDM (1977). Stereoscopic atlas of macular diseases: diagnosis and treatment.

[30] Ho AC, Yannuzzi LA, Pisicano K, DeRosa J (1995). The natural history of idiopathic subfoveal choroidal neovascularization. Ophthalmology..

[31] Cukras C, Agrón E, Klein ML, Ferris FL, Chew EY, Gensler G (2010). Natural history of Drusenoid pigment epithelial detachment in age-related macular degeneration: AREDS report number 28. Ophthalmology..

[32] Yehoshua Z, Wang F, Rosenfeld PJ, Penha FM, Feuer WJ, Gregori G (2011). Natural history of drusen morphology in age-related macular degeneration using spectral domain optical coherence tomography. Ophthalmology..

[33] Spaide RF, Ooto S, Curcio CA (2018). Subretinal drusenoid deposits AKA pseudodrusen. Surv Ophthalmol.

[34] Wilde C, Patel M, Lakshmanan A, Morales MA, Dhar-Munshi S, Amoaku WMK (2016). Prevalence of reticular pseudodrusen in eyes with newly presenting neovascular age-related macular degeneration. Eur J Ophthalmol.

[35] Marsiglia M, Boddu S, Chen CY, Jung JJ, Mrejen S, Gallego-Pinazo R (2015). Correlation between neovascular lesion type and clinical characteristics of nonneovascular fellow eyes in patients with unilateral, neovascular age-related macular degeneration. Retina (Philadelphia, Pa).

[36] Abugreen S, Muldrew KA, Stevenson MR, VanLeeuwen R, DeJong PTVM, Chakravarthy U (2003). CNV subtype in first eyes predicts severity of ARM in fellow eyes. Br J Ophthalmol.

