# Correction to: Drusen characteristics of type 2 macular neovascularization in age-related macular degeneration

**DOI:** 10.1186/s12886-020-01699-0

**Published:** 2020-10-27

**Authors:** Daniel Ahmed, Martin Stattin, Anna-Maria Haas, Alexandra Graf, Katharina Krepler, Siamak Ansari-Shahrezaei

**Affiliations:** 1 Karl Landsteiner Institute for Retinal Research and Imaging, Vienna, Austria; 2 Department of Ophthalmology, Rudolf Foundation Hospital, Juchgasse 25, 1030 Vienna, Austria; 3 Center for Medical Statistic, Informatics, and Intelligent Systems, Medical University of Vienna, Spitalgasse 23, 1090 Vienna, Austria; 4 Department of Ophthalmology, Medical University of Graz, Auenbruggerplatz 1, 8036 Graz, Austria

**Correction to: BMC Ophthalmol (2020) 20:381**

**http://orcid.org/10.1186/s12886-020-01651-2**

Following publication of the original article [1], we were notified that Fig. 1 was incorrect. Figure 1 was meant to picture Drusen ≥63 μm and SDD, however a duplicate of Fig. 3 was published. The correct Fig. 1 with its respective legend is displayed below.

**Fig. 1:**
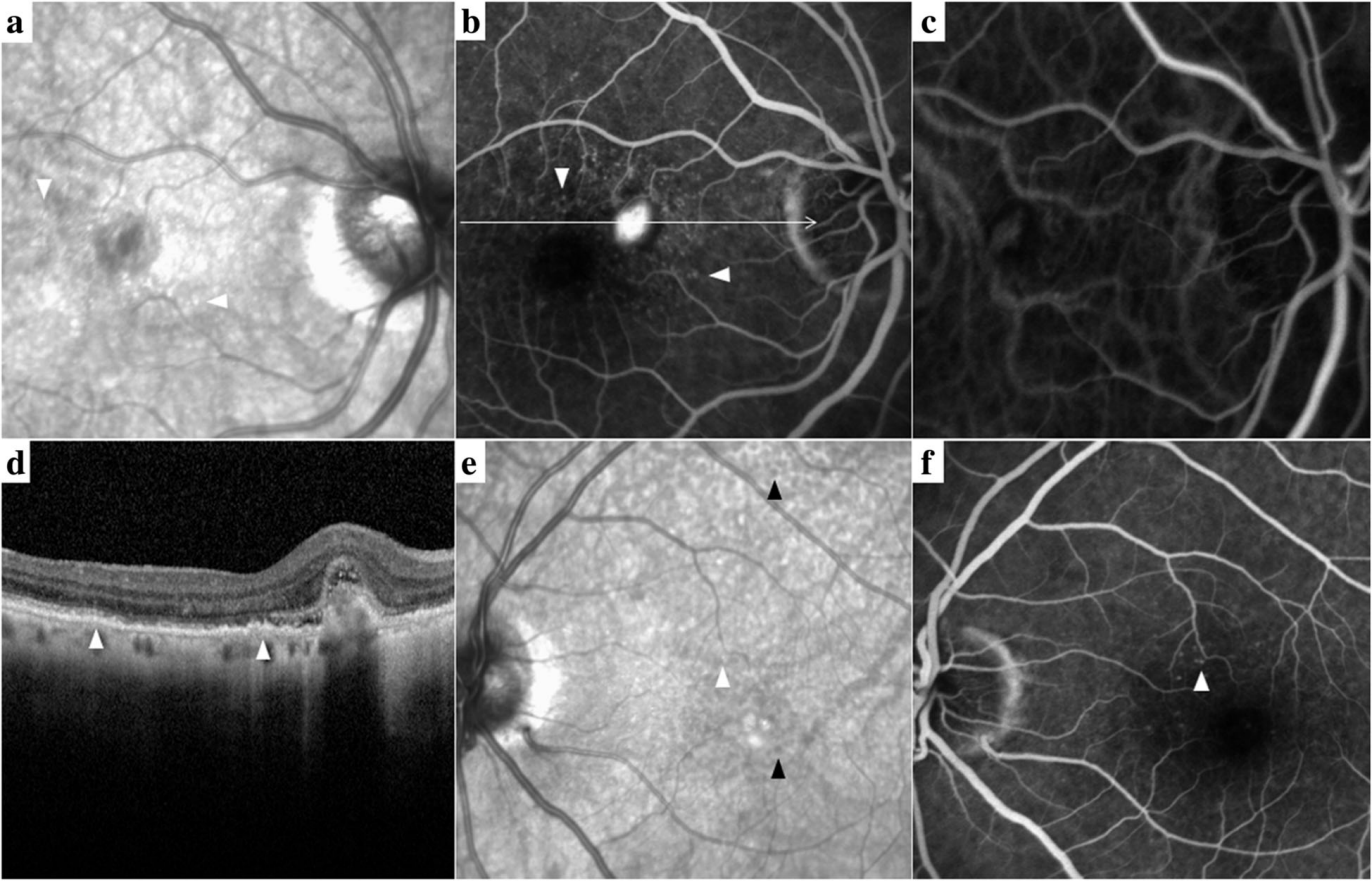
Multimodal imaging of type 2 MNV, Drusen ≥63 μm and SDD. **a** Drusen ≥63 μm (arrowheads) displayed in IR. **b** FA with early MNV leakage and drusen staining (scan line). **c** ICGA revealed a circumscribed MNV besides choroidal vessels under speckled hypocyansecence. **d** SD-OCT B-scan section through the lesion illustrated soft drusen (arrowheads) and a thin SFCT (109 μm). **e** Drusen > 63 (white arrowhead) and SDD (black arrowheads) visualized in the patient’s fellow eye as demonstrated in IR and **f** soft drusen staining in late FA
